# Young Aphids Avoid Erroneous Dropping when Evading Mammalian Herbivores by Combining Input from Two Sensory Modalities

**DOI:** 10.1371/journal.pone.0032706

**Published:** 2012-04-09

**Authors:** Moshe Gish, Amots Dafni, Moshe Inbar

**Affiliations:** Department of Evolutionary and Environmental Biology, University of Haifa, Haifa, Israel; Centro de Investigación y de Estudios Avanzados, Mexico

## Abstract

Mammalian herbivores may incidentally ingest plant-dwelling insects while foraging. Adult pea aphids (*Acyrthosiphon pisum*) avoid this danger by dropping off their host plant after sensing the herbivore's warm and humid breath and the vibrations it causes while feeding. Aphid nymphs may also drop (to escape insect enemies), but because of their slow movement, have a lower chance of finding a new plant. We compared dropping rates of first-instar nymphs with those of adults, after exposing pea aphids to different combinations of simulated mammalian breath and vibrations. We hypothesized that nymphs would compensate for the greater risk they face on the ground by interpreting more conservatively the mammalian herbivore cues they perceive. Most adults dropped in response to breath alone, but nymphs rarely did so. Breath stimulus accompanied by one concurrent vibrational stimulus, caused a minor rise in adult dropping rates. Adding a second vibration during breath had no additional effect on adults. The nymphs, however, relied on a combination of the two types of stimuli, with a threefold increase in dropping rates when the breath was accompanied by one vibration, and a further doubling of dropping rates when the second vibration was added. The age-specificity of the aphids' herbivore detection mechanism is probably an adaptation to the different cost of dropping for the different age groups. Relying on a combination of stimuli from two sensory modalities enables the vulnerable nymphs to avoid costly mistakes. Our findings emphasize the importance of the direct trophic effect of mammalian herbivory for plant-dwelling insects.

## Introduction

Juvenile animals are usually smaller and less agile than adults. As a consequence, young are often more vulnerable to attack by predators. Indeed, some predators take advantage of this and prefer to attack juveniles [1], [2]. This difference between juveniles and adults has led in many cases to the development of age-specific, passive and active defense strategies. For example, Thomson's gazelle fawns and young Iberian green frogs rely on crypsis more than adults, tolerating shorter approach distances of the predator before executing an escape response [3], [4]. In broad-headed skinks, on the other hand, the adults are the cryptic ones and the juveniles wave their brightly colored tails to deflect predators away from their body [5]. Another way of defending against predators is to display aggression. Adult American lobsters threaten and attack an approaching predator as opposed to the juveniles which prefer to retreat [6], [7]. In other animal species, the juveniles are the aggressors: some species of gall-forming aphids produce first or second-instar soldiers that defend the colony by clasping insect predators and piercing them with their stylets [8]; in several snake species, juveniles, which suffer greater predator-induced mortality, are more likely to display aggressive-defensive behaviors [9], [10]. Juveniles may also compensate for their higher vulnerability to predators by escaping, more frequently than adults, to a different part of their habitat where they are camouflaged or less accessible, as demonstrated in grasshoppers [11] and freshwater snails [12].

Aphids (Homoptera: Aphididae) are good candidates for studying behavioral differences between young and mature individuals, for several reasons: they are rapidly reproducing, sedentary herbivorous insects that form colonies of mixed ages [13]; they are subjected to a multitude of predators and parasitoids [14]; they possess an array of defensive behaviors. Aphids may defend against their insect enemies (namely ladybugs, hoverfly larvae, lacewings, parasitic wasps, etc.) by secreting a sticky defensive substance that adheres to the predator's mouthparts, kicking, twitching, walking away or dropping off the host plant [15]–[19]. Dropping is the most effective way of escaping from enemies on the plant, but it also exposes the aphid to the risks of dying from high ground temperatures, being preyed upon by ground predators, or failing to find a new host plant [20]–[23]. Even if an aphid is successful in locating a new host plant, its fecundity may be impaired due to the expenditure of energy on searching and the loss of feeding time. Roitberg et al. [21] found that on the day after the dispersal of pea aphids, *Acyrthosiphon pisum* Harris, to new host plants following insect-predator disturbance, their fecundity dropped almost two thirds. Nelson [24] estimated the reduction in pea aphid total fecundity the day after a single predator-induced dispersal event at about 20%. An aphid is therefore expected to drop only when the cost of staying on the plant becomes greater than the cost of dropping [16], [17].

Another important threat to an aphid colony is being consumed by mammalian herbivores along with their host plant (incidental ingestion). The incidental ingestion of plant-dwelling insects by mammalian herbivores is a direct interaction that has been practically ignored by ecologists. It is probably a very common interaction [25], yet only a handful of studies have examined its ecological significance [26], [27]. Incidental ingestion by mammalian herbivores could profoundly affect plant-dwelling insects, and in at least a few aphid species has led to the development of an efficient defensive behavior: upon sensing the warm and humid breath of a mammalian herbivore, the aphids instantaneously drop off the plant in large numbers. In this way most of the adult aphids in the colony avoid being eaten by the herbivore [28], [29].

In addition to exhaling air, large herbivores also cause vibrational disturbances when brushing against or tearing off pieces of the plant. Hence, vibrations may also contribute to the aphids' mass dropping response [28], [29]. Substrate-borne vibrations have been shown to serve as indication to an approaching predator and to elicit an evasive dropping response in aphids [30], [31] and other animals. For example, larvae of a geometrid moth escape by hanging from silk threads when sensing the vibrations produced by insect enemies [32]. Embryos of the red-eyed treefrog hatch up to 30% earlier and drop from overhanging vegetation to the water, upon sensing the vibrations induced by egg-eating snakes [33]. The antipredator response of pea aphids increases when a simulated predator attack is composed of two cues: alarm pheromone secreted by conspecifics and vibrations [30]. The role of vibrational stimuli in the escape of aphids from mammalian herbivores, and the interplay between the response to mammalian breath and the response to vibration is, however, still unclear.

The cost of dropping off the plant is higher for young nymphs than it is for adults, because nymphs are more limited in their ability to walk and locate a new host plant [34] and are more susceptible, after dropping, to high air and ground temperatures than adults are [20], [23], [35]. Tokunaga and Suzuki [36] found that first-instar pea aphid nymphs walk, on average, 8 times more slowly than adults. Roitberg et al. [21] examined the dispersal of pea aphids to new host plants after escaping from ladybug attack, and found that first and second instar nymphs were 5 times more likely than apterous adults to die on the ground before reaching a new host plant. They also found that apterous adults were twice as likely to disperse to a new host plant as first and second instar nymphs, who tend to return to the original host. Due to the high cost of dropping, young nymphs are often less likely than adults to respond to a predator or parasitoid attack by dropping off the plant [17], [34], [37].

According to the threat-sensitive predator avoidance hypothesis [38], prey animals assess the risk of predation they perceive, and modulate their antipredator responses according to the level of risk. Presumably, this allows prey to balance the cost of predator avoidance with the danger of being caught.

We therefore hypothesized that because of the higher cost of dropping for the nymphs, they would require a more definitive indication of impending mammalian herbivory than would be needed by adults. Gish et al. [28], [29] have described the mass dropping of aphids in response to mammalian herbivore feeding, but have focused only on the behavior of adult aphids. In the current study we exposed pea aphids to simulated mammalian breath and to vibrational disturbance caused by automated leaf-picking. We quantified and compared the dropping responses of first-instar nymphs (henceforth referred to as “nymphs") and adults to different combinations of the two types of stimuli.

## Materials and Methods

### Experimental plants and animals

Pea aphids were reared on broad bean plants, *Vicia faba* L., that were planted in plastic cups filled with a commercial growing medium and kept in the laboratory at 22°C±1°C, 65%±10% relative humidity (RH) and a photoperiod of 16∶8 L∶D. Broad bean plants were used in the experiments when they were 13±1 days old (∼15 cm tall, having two fully developed compound leaves). All aphids were descendants of a single parthenogenetic female collected at Kiryat-Tivon, Israel. Each aphid and each plant was used for experimentation only once. We used apterous (non-winged) aphids in all experiments. Throughout the research, room temperature was kept at 22°C±1°C and RH was 67%±7%.

### Experimental setup

We conducted a series of experiments that included different combinations of simulated mammalian herbivore breath and leaf-picking vibrations.

**Mammalian breath simulation:** We simulated mammalian breath using an artificial breath apparatus (detailed description in [28]). Briefly, the apparatus creates a steady airstream at desired velocity, temperature and humidity by bubbling a stream of filtered air through water at a fixed temperature (air velocity 4 m×s^−1^). The apparatus was adjusted so that, in all experiments, the airstream's temperature and humidity were 35.5°C±0.5°C and >90% RH, respectively (similar to the temperature and humidity of typical mammalian breath). The airstream flowed out of an insulated flexible silicone hose that was pointed at the plant's apex from a distance of approximately 2 cm. Carbon dioxide has no effect on pea aphid dropping behavior [28] and therefore we did not manipulate its concentration in the airstream.

**Figure 1:**
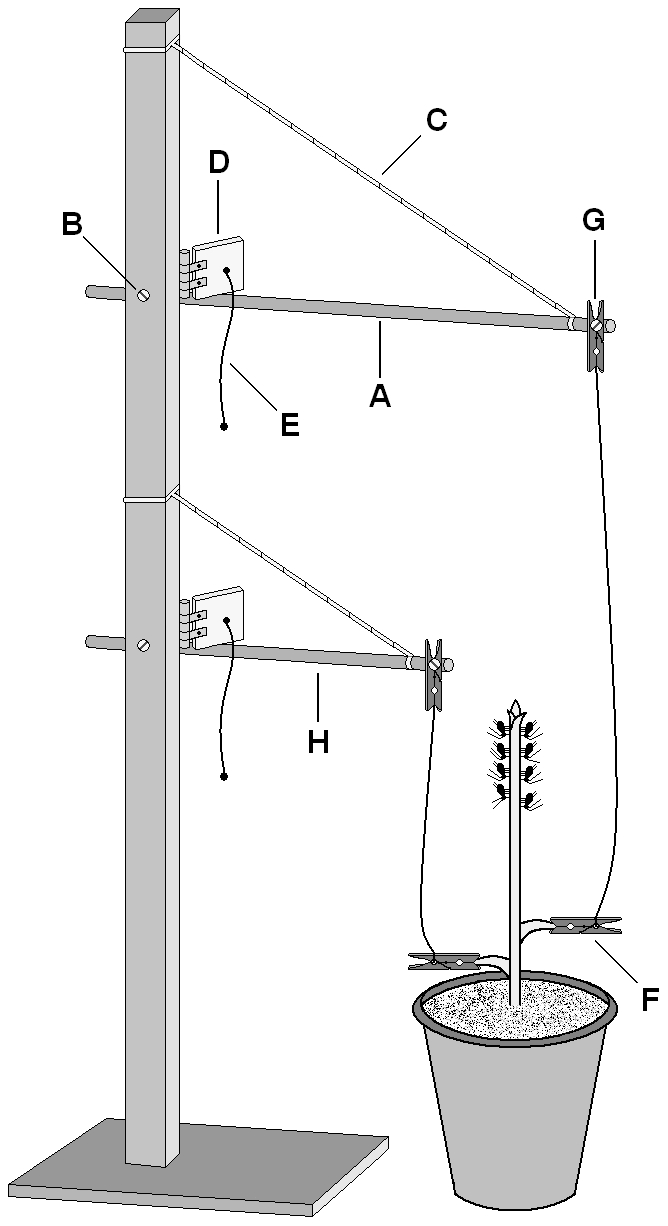
Description of the leaf picking device. A lever (A) is connected on one end to an upright post, so that it is free to rotate around the connection point (B). The distal end of the lever is connected to the post with a rubber band (C). The lever is lowered so that it is perpendicular to the post and the rubber band is taut. The lever is held in place with a moving stop (D). A small clothespin (F) is attached to a juvenile leaf at the base of a broad bean stem. A string is tied at one end to the clothespin (F), and at the other end attached to another clothespin (G) which is connected to the distal end of the lever. A slight pull on the string that is attached to the stop (E) releases the lever, allowing it to spring up and tear off the leaf. A second lever (H) is constructed in the same way. In the experiments that included two vibrations, first the lower lever (H) was released and then the upper lever (A). The device drawn here is in a “ready for operation" state at the beginning of an experiment.

**Leaf-picking vibrations:** We built a leaf picking device that simulated the vibration caused by a feeding mammalian herbivore (Fig. 1). The device was designed to pick a leaf off a broad bean plant with the pull of a trigger. The evening before an experiment, each broad bean plant was stripped of all compound leaves for convenience, leaving only the apical bud and two juvenile leaves growing at the bottom of the stem (broad bean plants typically have two small alternate juvenile leaves that precede the growth of the compound leaves). A small 3 cm long clothespin paper holder with a connected string was attached to each of the two juvenile leaves (Fig. 1F). Approximately 15 adult aphids were then placed on each stem. Pea aphids (both nymphs and adults) tended to aggregate on the apexes, although some moved about and sometimes left the plant during the night. The following morning, each plant had adult aphids that remained on the stem and nymphs that were born during the night. The average number of adults and nymphs on the upper third of each stem was 10.6±2.1 and 71.2±16.5 (SD), respectively.

Thirty to ninety minutes before the beginning of each trial, both strings were raised and carefully attached to the two clothespin paper holders at the distal ends of the levers (Fig. 1G). As the strings were kept slightly loose and handling was done very carefully, this procedure caused no visible vibrations to the plant and no observable disturbance to the aphids. All work near the aphids was done while the researcher (MG) was wearing a surgical mask and holding his breath, to minimize the disturbance to the aphids. The experiment took place on the concrete-based floor of the laboratory, to prevent the vibrations caused by the operation of the leaf picking device from affecting the aphids on plants that were not yet tested. The precise timing of the trigger pulling and of the artificial breath application was done to the beat of a metronome (1 beat×s^−1^). After the application of the tested stimuli, aphids that dropped were counted. The most convenient way of counting the aphids that remained on the plant was by removing them, which most likely caused them to release alarm pheromone. In order to lower the exposure of aphids that were not yet tested to alarm pheromone which may affect their behavior, the act of removing the aphids and counting them was performed in a separate room. In addition, the laboratory was aired for a minimum of 45 min between trials.

Because of variation in vibration intensity along the stem and the limited diameter of the artificial breath plume, only aphids that were situated on the upper third of the stem were included in the experiments. This was done by counting the adults and nymphs that were on the bottom two thirds of the stem prior to the beginning of each trial. When a trial ended, the number of aphids that were absent from the bottom two thirds of the stem was subtracted from the total count of aphids found on the floor or in the pot. To ensure uniformity, each trial was started when none of the aphids on the plant were moving, or in the process of giving birth.

### Experimental design

**Figure 2:**
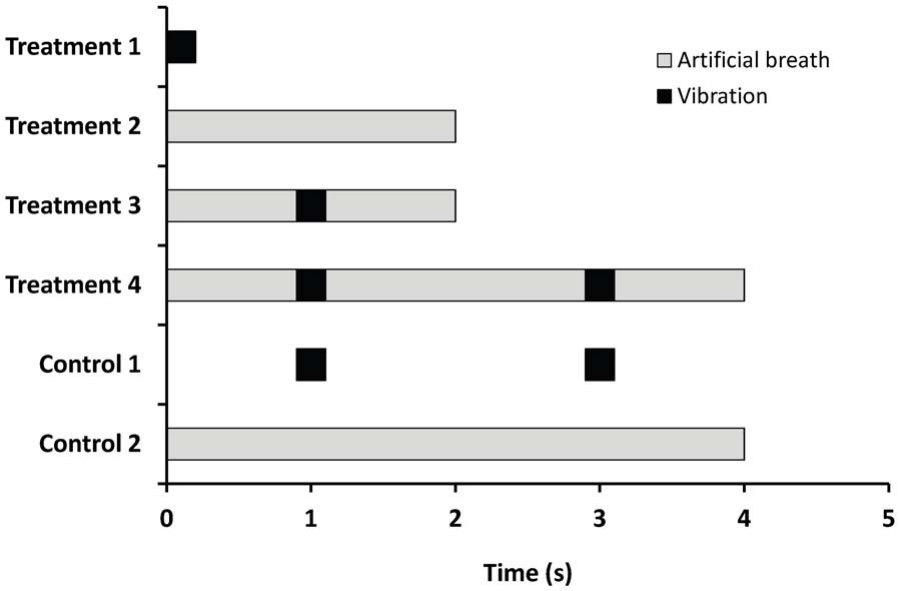
Examination of pea aphid dropping response to different stimuli (and their combinations): details of experimental design. The X axis denotes the time from the beginning of the experiment. In all treatments and controls N = 20.

We examined the response of both nymphs and adults to the following combinations of stimuli: a single vibration; a 2 s artificial breath; vibration and breath applied simultaneously; and two consecutive vibrations applied simultaneously with breath. The latter treatment required a longer breath of 4 s. A specification of the combination of cues used in each treatment and the way they were combined is given in Fig. 2. We also designed two control tests to be performed in the case of a rise in dropping when a second vibration is added (a significant difference between treatments 3 and 4, see Fig. 2). Such a rise could be caused by the intensification of only one stimulus type (i.e. lengthening the breath of adding a second vibration), or by the combined effect of the two stimulus types. In order to check for a possible independent influence of adding a second vibration, we examined the response to two consecutive vibrations, with no breath (control 1). In order to check for an independent influence of the elongated duration of the breath (4 s in treatment 4, as opposed to 2 s in treatment 3, see Fig. 2), we examined the response to a breath that lasted 4 s, with no vibration (control 2). Each treatment was replicated 20 times.

### Statistical analyses

Data sets were arcsin square-root transformed and checked for normality and homogeneity of variances using the Shapiro-Wilk's test and Levene's test respectively. Data from treatments 2–4 (see Fig. 2) were analyzed using a two way analysis of variance (ANOVA), with aphid age (nymphs or adults) and stimuli (2 s breath, 1 vibration during a 2 s breath, 2 vibrations during a 4 s breath) as fixed factors.

For each age group, when the addition of a second vibration caused a significant rise in dropping rates, the following three values were analyzed using a one way ANOVA with post hoc comparisons (Tukey's HSD): 1. The difference between the results of treatment 1 (a single vibration without breath) and the results of control 1 (two consecutive vibrations without breath). 2. The difference between the results of treatment 2 (2 s breath without vibration) and the results of control 2 (4 s breath without vibration). 3. The difference between the results of treatment 3 (one vibration and a 2 s breath applied simultaneously) and the results of treatment 4 (two consecutive vibrations applied during a 4 s breath).

In order to use the differences between the results of two treatments in the analysis of variance, we arranged the data from the two treatments in random pairs and produced a set of 20 data from the differences within the pairs. All statistical analyses were conducted using SPSS (Version 15).

## Results

A single vibration (treatment 1) caused practically no dropping both in nymphs and adults. On average (± SE) only 0.3%±0.1% of the nymphs and 5%±2.3% of the adults in the colony dropped in response to this treatment.

**Figure 3:**
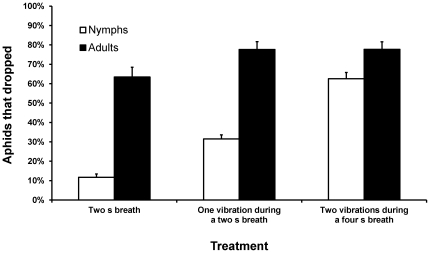
Response of pea aphids (*Acyrthosiphon pisum*) to artificial breath and to vibration caused by a leaf picking device. Error bars represent standard errors (±1 SE). In all treatments N = 20.

Nymphs, however, did differ from adults in their response to simulated mammalian breath and to the combination of breath and vibration. While most adults dropped when exposed to the 2 s warm and humid airstream (treatment 2), nymphs showed only a mild dropping response. Adding one vibration to the breath stimulus (treatment 3) increased by threefold the dropping rate of nymphs, but only slightly affected the adults. Addition of a second vibration during the application of the breath stimulus (treatment 4) caused a further doubling of nymph dropping rates (an overall rise of six times the dropping rates caused by breath alone), but almost no change in adult dropping rates (an increase of less than 0.5%) (Fig. 3; Table 1).

**Table 1:** Two way ANOVA of the effects of “Age" and “Treatment" on pea aphid dropping response.

Source	df	Mean Square	F	Sig.
Age	1	6.41	136.6	*P*<0.001
Treatment	2	1.44	30.65	*P*<0.001
Age×Treatment	2	.49	10.50	*P*<0.001
Error	114	.05		

The two age groups were: First-instar nymphs and adults. The three treatments were: A. Two s breath. B. One vibration during breath. C. Two vibrations during breath.

**Figure 4:**
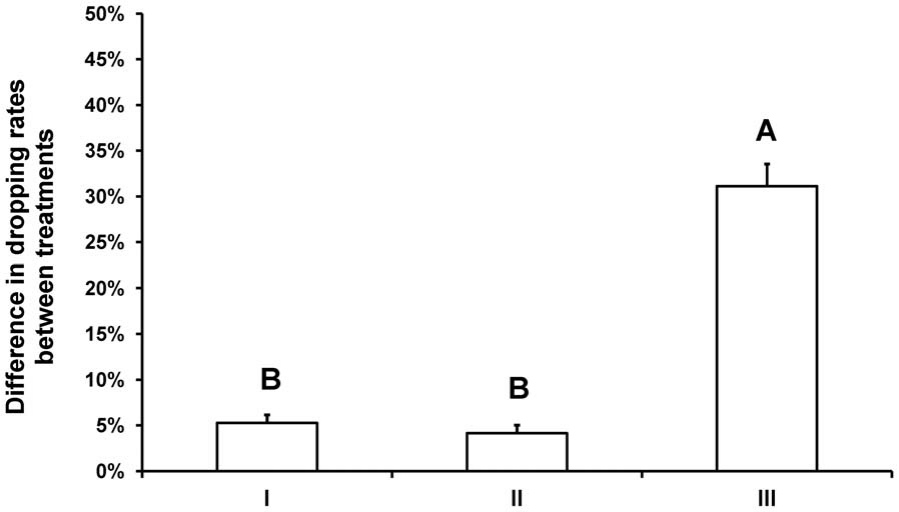
Increase in the dropping rates of pea aphid nymphs when exposed to a second vibration and its two controls. Data are the average percentage of the nymphs in the colony that dropped (±1 SE). Percentage data were arcsine square-root transformed prior to analysis. X-axis labels denote: I: The difference between the response to a 2 s and a 4 s artificial breath. II: The difference between the response to one vibration and two consecutive vibrations. III: The difference between the response to one vibration during a 2 s artificial breath and two vibrations during a 4 s artificial breath. Each bar represents an average of the differences within 20 randomly assigned pairs of data from the two compared treatments.

For this reason we performed controls 1 and 2 with nymphs only (see experimental design and Fig. 2). In control 1, two consecutive vibrations caused 4.3%±0.9% of the nymphs to drop. In control 2, a 4 s breath caused 13.8%±1.5% of the nymphs to drop. One-way ANOVA (F_2,57_ = 83.45, P<0.001, Fig. 4) ruled out the possibility that the doubling of dropping rates of the nymphs when a second vibration was applied was caused by the independent action of prolonging the breath or intensifying (i.e. two stimuli instead of one) the vibrational stimulus. See the statistical analyses section in the materials and methods for an explanation on the one-way ANOVA.

## Discussion

We found that pea aphid nymphs have a different tipping point from adults when dropping in response to cues that are typical of mammalian herbivore feeding; most of the adults escape incidental ingestion upon sensing the herbivore's breath alone, while nymphs tend to drop only when sensing concurrent breath and vibrational stimuli (Fig. 3). Vibrations caused by leaf picking are an indication that part of the plant was just eaten by an herbivore, but they do not necessarily mean that the plant part on which the aphid is situated will be eaten next. This could be the reason that the aphids (nymphs and adults) rarely drop in response to the vibrational stimulus alone (treatment 1). A warm and humid breath (treatment 2) on the other hand, is a reliable indicator of a close herbivore snout, although it is not a sure sign of impending incidental ingestion since the herbivore may exhale in the aphid's direction while feeding on other parts of the plant. Adult aphids mostly regard the breath as sufficient warning whereas most nymphs stay on the plant, waiting for further verification of the imminent danger. Leaf picking vibrations combined with breath (treatment 3) are probably an indication that an herbivore has just fed on a very close plant part. The chances of an aphid being eaten in this case probably rise significantly, so that a third of the nymphs choose to escape. A second vibration (treatment 4) seems to further verify this conclusion, raising nymph dropping rates to two thirds. The two controls we performed prove that the high dropping in response to the second vibration depended on the perception of the other two stimuli (breath+first vibration).

The force of the airstream in the breath treatments is unlikely to be the cause for the aphids' dropping, for aphids are not easily dislodged by wind, even on very windy days [29]. The air velocity used here (4 m/s) was identical to the air velocity that, when at room temperature and humidity, caused no dropping in previous studies [28], [29]. Furthermore, it is improbable that the leaf picking vibration loosened the aphids' grip, causing them to be dislodged by the force of the airstream. If the leaf picking vibration, which vigorously shook the plant, was to loosen the aphids' grip, there should have been substantial dropping with the application of the second vibration in control 1. Nevertheless, in control 1 the dropping rate was only 4.3%.

The differential nature of the aphids' evasive response is consistent with the threat-sensitive predator avoidance hypothesis, which predicts that prey animals will adjust the intensity of their predator avoidance behavior to the degree of perceived predatory threat [38]. Relying on a combination of two cues to execute a defensive response increases the accuracy of the detection mechanism by minimizing the chance of mistake. This is of great importance when the cost of an erroneous defensive response is high.

A similar conservative double-stimulus threat detection mechanism has been reported in lotic ecosystems (running water), where the escape of aquatic insects from insect predators may expose them to fish predators [39] or increase their risk of drifting downstream and losing foraging opportunities. Mayfly nymphs (Ephemeroptera) crawl or swim away to evade predatory stonefly nymphs (Plecoptera). Nymphs of some mayfly species respond to the tactile stimulus of the predator more often when in the presence of stonefly chemical cues, presumably to minimize the chances of an unnecessary departure from a food patch [40].

Dependence on the perception of two different stimuli is also found in a species of crayfish, which responds to a visual predator stimulus from a greater distance and retreats further when exposed to the scent of an injured conspecific [41]. In a similar manner, juvenile Atlantic salmon take longer to resume foraging when a visual predator stimulus follows the exposure to injured conspecific scent [42].

The use of conservative, double-stimulus threat detection mechanisms can also be found in terrestrial habitats. Wall lizards escape attacks from birds and mammals by hiding inside rock crevices. These rock crevices are sometimes inhabited by snakes that feed on lizards seeking refuge. It is therefore critical for wall lizards to accurately assess the probability of an ambush inside a rock crevice, because overestimating the risk would cause the lizards to remain exposed and vulnerable. Amo et al. [43] showed that when a refuge contains both the scent and the image of a snake, wall lizards depart from it earlier.

The principle of executing a response when perceiving more than one stimulus is not restricted to defense mechanisms and is sometimes implemented in other costly activities. In the carnivorous plant “Venus flytrap", the inner surface of the trap contains several modified hairs that function as touch sensors. The trap snaps closed only when one or more of the trigger hairs are mechanically stimulated twice within a period of 25 seconds [44], [45]. The requirement for two stimuli lowers the chances of the trap being activated by random mechanical stimuli instead of a live insect, which would result in a waste of energy [46] and loss of feeding opportunities until the trap resets.

When used for defense, the dependence on a combination of cues may come with a cost: waiting for a second stimulus to appear, shortens the time available for the animal to defend itself. In addition, if one of the stimuli is absent, the animal may not be able to employ its defense. Apparently, for adult pea aphids these disadvantages outweigh the advantage of minimizing the chance for mistake. This could be the reason they do not rely on a double-stimulus mechanism to detect approaching herbivores. For nymphs, however, the advantage of increased accuracy outweighs the disadvantages. This difference between nymphs and adults is probably an adaptation to differences in performance on the ground: while nymphs have a high chance of mortality after dropping off the plant (see introduction), adult pea aphids are highly mobile on the ground and capable of walking and settling on distant host plants. We have tracked adult apterous pea aphids marked with fluorescent powder that were released in a meadow, and found them 8 hours later on host plants located up to 10 m away (Gish et al., unpublished).

It should be noted that when aphids drop from their host plant, they don't necessarily reach the ground, as was assumed in many studies [22], [47], [48]. After dropping though, they might land on lower parts of the same plant, on other adjacent plants or on plant litter. Nelson [24] noted that the pea aphids in his study rarely contacted the ground after dropping from alfalfa in response to a predator. Even if an aphid doesn't reach the ground itself, it may still pay a high reproductive cost for the loss of feeding time and energy or fail to find a new suitable host. It is therefore probable that the cost of dropping is higher for young nymphs even if they don't reach the ground after dropping.

Dropping off the host plant, the most dramatic and costly defensive behavior in aphids, is reserved for situations when the danger in staying on the plant is greater than the dangers faced on the ground. Accurate risk detection is therefore essential for this behavior to be selected. The use of the mass dropping behavior by pea aphids is optimized by adjustment of the sensitivity threshold according to age and by the utilization of a double-stimulus mechanism.

The plants that we used in our study were mechanically damaged (leaves removed) before they were used for experimentation (see materials and methods). The plants were therefore most likely to release damage-induced plant volatiles, which may directly affect the behavior of herbivorous insects, including aphids [49]–[51]. In our study, if such an effect existed, it was unlikely to bias the interpretation of the results, since all plants in all treatments and controls received the same mechanical damage. Furthermore, a previous study [28] found that the mass dropping response in pea aphids occurs on undamaged plants. Nevertheless, it is possible that the exposure of aphids to damage-induced plant volatiles brought them into an ‘alerted’ phase, in which they were more responsive to the triggers that we used. It would be interesting to examine, in future studies, whether herbivore-induced plant volatiles that are released following mammalian feeding influence aphids' ability to escape from mammalian herbivores.

Our research describes a threat detection mechanism in one genotype of pea aphids. It is not unlikely that other genotypes behave differently, as pea aphids are known to have considerable genetic variation among populations [52]. Very often, pea aphids from different habitats and host plants differ in their defensive responses, including their propensity to drop off their host plant [20], [53]. It would be interesting to examine the existence of the double-stimulus threat detection mechanism in other pea aphid races and in other aphid species. Such variation would provide insight into the interaction between mammalian and insect herbivores in different habitats and on various host plants.

The double-stimulus mechanism is analogous to similar adaptations found throughout nature, where high costs of employing important responses favored the reliance on more than one stimulus, and differences in vulnerability between juveniles and adults have led to the development of different defense strategies.

The existence of specialized defense mechanisms, protecting aphids from incidental ingestion, points to the importance of mammalian herbivory for plant-dwelling insects. Additional ecological research that will link anti-herbivore defense mechanisms with insect population dynamics is likely to shed more light on the way mammalian herbivory shapes plant-dwelling insect communities.
